# Cerebrospinal Fluid Proteome Alterations Associated with Neuropsychiatric Symptoms in Cognitive Decline and Alzheimer’s Disease

**DOI:** 10.3390/cells11061030

**Published:** 2022-03-18

**Authors:** Magdalena Mroczek, Christopher Clark, Loïc Dayon, Gene L. Bowman, Julius Popp

**Affiliations:** 1 Department of Geriatric Psychiatry, University Hospital of Psychiatry Zürich, University of Zürich, 8032 Zürich, Switzerland; m.mroczek888@gmail.com; 2 Institute for Regenerative Medicine, University of Zürich, 8952 Schlieren, Switzerland; christopher.clark@irem.uzh.ch; 3 Nestlé Institute of Food Safety & Analytical Sciences, Nestlé Research, 1015 Lausanne, Switzerland; loic.dayon@rd.nestle.com; 4 Institut des Sciences et Ingénierie Chimiques, Ecole Polytechnique Fédérale de Lausanne, 1015 Lausanne, Switzerland; 5 Nestlé Institute of Health Sciences, Nestlé Research, 1015 Lausanne, Switzerland; drgenebowman@gmail.com; 6 NIA-Layton Aging and Alzheimer Disease Center, Department of Neurology, Oregon Health & Science University, Portland, OR 97239, USA; 7 Helfgott Research Institute, National University of Natural Medicine, Portland, OR 97201, USA; 8 Old Age Psychiatry, Department of Psychiatry, University Hospital of Lausanne, 1012 Lausanne, Switzerland

**Keywords:** proteome, Alzheimer’s disease, cognitive decline

## Abstract

Although neuropsychiatric symptoms (NPS) are common and severely affect older people with cognitive decline, little is known about their underlying molecular mechanisms and relationships with Alzheimer’s disease (AD). The aim of this study was to identify and characterize cerebrospinal fluid (CSF) proteome alterations related to NPS. In a longitudinally followed-up cohort of subjects with normal cognition and patients with cognitive impairment (MCI and mild dementia) from a memory clinic setting, we quantified a panel of 790 proteins in CSF using an untargeted shotgun proteomic workflow. Regression models and pathway enrichment analysis were used to investigate protein alterations related to NPS, and to explore relationships with AD pathology and cognitive decline at follow-up visits. Regression analysis selected 27 CSF proteins associated with NPS. These associations were independent of the presence of cerebral AD pathology (defined as CSF p-tau181/Aβ1–42 > 0.0779, center cutoff). Gene ontology enrichment showed abundance alterations of proteins related to cell adhesion, immune response, and lipid metabolism, among others, in relation to NPS. Out of the selected proteins, three were associated with accelerated cognitive decline at follow-up visits after controlling for possible confounders. Specific CSF proteome alterations underlying NPS may both represent pathophysiological processes independent from AD and accelerate clinical disease progression.

## 1. Introduction

Neuropsychiatric symptoms (NPS) are present in the majority of the patients with probable Alzheimer’s disease (AD) and other dementias [1]. NPS are debilitating to quality of life, come with a caregiver burden, create cognitive and functional impairment, have a poor prognosis, i.e., disease progression, and result in earlier institutionalization and increased mortality. NPS may start very early in the course of cognitive decline leading to dementia [2]. Of all NPS, depression is the most frequently observed syndrome in people with mild cognitive impairment (MCI) and early dementia due to AD [3]. The five-year period prevalence was found to be highest for depression, apathy, and anxiety [4]. Agitation, delusions, and hallucinations become more common in advanced disease stages, whereas apathy is the most persistent and frequent NPS throughout all stages of AD [5]. Anxiety has been found to occur early and to be a risk factor for AD dementia [6].

Although NPS have major clinical consequences for those affected, their caregivers, and the healthcare systems, the specific underlying pathophysiological mechanisms are unclear [7]. There is a need to characterize the molecular profiles and pathway alterations related to NPS in relation to cognitive decline and AD [8]. In subjects with AD, different pathological processes in the brain may result in NPS, and these may be related or not to the core AD pathology. Finally, early diagnosis of NPS-related processes, which can often precede the first symptoms of dementia and MCI, is crucial in implementing appropriate therapeutic approaches to both reduce symptoms’ intensity and slow down clinical progression of the disease [9].

Several studies addressed associations of NPS with biomarkers of the core AD pathology. Most studies found associations of core AD biomarkers (increased CSF tau and p-tau levels, decreased CSF amyloid levels, or a combination of them) with NPS [10]. It remains unclear, however, whether the pathophysiological alterations underlying NPS are closely related to, or independent of the presence of AD pathology. Although some efforts have been made to study the role of proteins other than biomarkers of AD pathology in NPS, the previous studies investigated only a very limited number of targeted molecules [11].

In recent years, new methods, such as the omics technologies, revolutionized the investigation of molecular alterations in disease, allowing for the untargeted assessment of a large number of molecules and the unbiased detection of changes related to clinical diagnosis and syndromes. These approaches, including metabolomics and proteomics, have been successfully applied in studies on cognitive decline and AD. However, studies on CSF alterations related to NPS and based on untargeted proteomics have not been published, yet.

The goal of our study was to determine the CSF proteome profiles of NPS in older adults with normal cognition and with cognitive impairment (mild cognitive impairment (MCI), and mild dementia) in a memory clinic setting. We hypothesized that specific proteome and biological pathway alterations are associated with NPS in general, and depression, apathy, and anxiety in particular. Additionally, we explored the relationships of the identified protein profiles with the presence of AD pathology as indicated by CSF biomarkers, and with faster cognitive decline over time.

## 2. Materials and Methods

### 2.1. Study Population

The study population consisted of 87 community dwelling individuals aged 53 to 85. They were recruited into a brain aging study conducted in the Department of Psychiatry and the Department of Clinical Neurosciences, University Hospital of Lausanne, Switzerland. The study was approved by the local ethics committee of the canton Vaud, Switzerland (No. 171/2013). Informed consent was obtained from all participants or their legal representatives. Cognitively impaired participants were recruited from memory clinic outpatients and did not present any major psychiatric or neurological disorders that may have affected the cognitive performance, nor substance abuse or severe or unstable physical illness. All subjects underwent a full clinical examination, made by senior neurologists, old age psychiatrists or geriatricians, and structural brain imaging (MRI or CT) [12]. The final diagnosis was reached based on the consensus group of clinicians. They met the diagnostic criteria for MCI [13] or mild AD dementia [14]. Cognitively healthy participants were recruited through journal announcements or word of mouth and did not present any medical conditions and no psychiatric or neurological disease clinically relevant for cognitive performance. They were assessed with the same procedure as patients (neuropsychological and neuropsychiatric assessment described in the study procedures, clinical examination, and imaging) and evaluated by a consensus group of clinicians (senior physicians and neuropsychologists). We previously performed proteomics in a larger number of participants [15]. All participants with available NPI-Q scores from this previous study were included in the current one.

### 2.2. Study Procedures

#### 2.2.1. Neuropsychological Cognitive and Neuropsychiatric Assessments

In this cross-sectional study an overall clinical, neurological, and comprehensive neuropsychological assessment and the administration of informant questionnaires were performed at baseline for all participants as previously described [16]. Briefly, performance in global cognition and cognitive status, and disease severity were assessed with the Mini Mental State Examination (MMSE), Clinical Dementia Rating (CDR), and CDR Sum of Boxes (CDR-SoB). Neuropsychiatric symptoms were assessed with the Neuropsychiatric Inventory Questionnaire (NPI-Q) [17]. The questionnaire includes ten behavioral neuropsychiatric disorders (delusions, hallucinations, agitation and aggression, dysphoria, anxiety, euphoria, apathy, disinhibition, irritability and lability, and aberrant motor activity) and two neurovegetative domains (nighttime behavioral disturbances, and appetite and eating changes), all scored on a 3-point scale according to severity. NPI-Q questionnaire has been filled in by the participant’s relative. The total NPI-Q score was determined by adding the twelve domain severity scores. Participants with a total NPI-Q score of >0 were considered NPS positive (i.e., having NPS). Participants with a positive score (>0) for depression were considered depression positive. The same group categorization was also applied for the apathy and anxiety domains. Additionally, the enrolled individuals were assessed with the following scales: the Buschke Double Memory Test, the digit span forward and backward, the Stroop Test, the letter fluency task, and the Trail Making Tests A and B. The functional assessment included the activities of daily living (ADL) and instrumental ADL (IADL). The clinical examination, the neuropsychological test battery, ADL, and IADL were used to determine the CDR and the CDR-SoB scores and to verify inclusion and exclusion criteria. All tests and scales used in this study are validated and widely used in the field [12]. Both CSF collection and NPI were assessed at baseline. The neuropsychological cognition tests were administered at baseline as well as at the follow-up (FU) visits. Clinical and neuropsychological FU evaluations were performed roughly every 18 months using the same methods and tests. The mean time to the last FU was 42 months.

#### 2.2.2. Sample Collection and Handling

Lumbar punctures yielding 10–12 mL of CSF were performed at baseline after an overnight fast in the memory center. Samples were spun down at 4 °C, immediately aliquoted, and snap frozen at −80 °C until assayed.

#### 2.2.3. CSF AD Biomarkers, Proteomics, and Apolipoprotein E Genotyping

CSF beta-amyloid 1–42 (Aβ1–42), total tau, and tau phosphorylated at threonine 181 (p-tau181) concentrations in the CSF samples were measured by ELISA with commercially available kits (Fujirebio, Gent, Belgium). Definition of having cerebral AD pathology or a CSF AD status was based on the positive AD CSF status defined a priori as CSF p-tau181/Aβ1–42 > 0.0779 as previously described [12]. Briefly, this value optimized group separation was based on the Youden index in a previous study using center data. Apolipoprotein E (APOE) genotype was determined through PCR testing as described before [12]. The positive carrier status was defined by the presence of at least one APOE ε4 allele.

CSF samples were measured using an untargeted shotgun proteomic workflow based on liquid chromatography (LC) and tandem mass spectrometry (MS/MS) [18]. The analyses were performed with an Ultimate 3000 RSLC nano system and a hybrid linear ion trap-Orbitrap (LTQ-OT) Elite (Thermo Scientific, San Jose, CA, USA). Relative quantification of proteins between the samples was performed using isobaric tagging with the tandem mass tag technology. This method provides relative fold-changes per protein for each sample with a mean standard deviation of the fold change of 0.2080 per protein. A total of 790 proteins was investigated (Supplementary Materials Table S1). Data acquisition and processing was previously described [15]. Of note, protein data were log_2_ -transformed to approach normality. The performing personnel was blinded to clinical data.

#### 2.2.4. Statistical Analysis

Before statistical analysis, outliers (i.e., data points that exceeded the cutoff value of mean ± 3 × SD) were replaced by the mean value. Normal distribution of protein measurements was assessed with Shapiro–Wilk test. Descriptive statistics were performed using the Mann–Whitney U-Test comparing NPS positive and negative groups for continuous variables and Chi-square tests for categorical variables. In the U-Test we assumed not normal distribution. To control for possible type 1 errors, U-Tests were also performed with an increased confidence interval (to 99%). The same group differences were observed in this case. Correlations between NPI-Q total score and proteins were assessed with Spearman’s rho. Statistical data analysis was performed with IBM SPSS statistics software version 25 (IBM Corporation, Armonk, NY, USA) and R software (version 3.6.1, Vienna, Austria). All statistical models were verified for possible overfitting using the Hosmer–Lemeshow test for goodness-of-fit. Models with a Hosmer–Lemeshow chi-squared value yielding a *p*-value > 0.05 were rejected and the previous iteration was considered instead.

In order to avoid saturation of the model (i.e., selecting only one variable from a group of inter-correlated variables) while keeping all variables in the model, we used Elastic-Net (EN) regularization for regression analysis and protein selection for NPI-Q > 0 for total score and for the depression, apathy, and anxiety domains. The NPI-Q > 0 was used as endpoint and associated features were identified using a value of λ (lambda) that minimized the 10-fold cross-validated error. We repeated this analysis, considering the presence of CSF AD status as a covariate. This was performed in the whole cohort using custom routines implementing the glmnet package [19]. These regression analyses do not consider interaction between variables but only their relative importance. To further reduce the number of proteins and build predictive models, independence of proteins for NPI-Q > 0 was tested with variance inflation factor (VIF) for NPI-Q > 0. Features with VIF > 10 were removed. This was not performed for proteins selected for apathy and depression as no diagnostic model was built for these domains. Resulting features were then used to construct a binary logistic regression model with NPI-Q > 0 or NPI-Q = 0 as the dependent variable. We considered sex, age, and cognitive performance (MMSE score) as confounders. In addition, we considered two diagnostic models, either including or excluding the presence of AD pathology as a variable to avoid bias caused by putatively strong correlation of AD with NPS. To select the best predictive models, we used an iterative approach, first adding all remaining proteins to a reference model considering sex, age, and cognitive performance (MMSE score) as confounders, and selected the model displaying the smallest Akaike information criterion (AIC) value to select the best molecule to add at each iteration. We repeated this process over successive iterations, adding a single analyte each time. Performance of the models was analyzed by comparing area under the curve (AUC) of the resulting receiver operating characteristic (ROC) curves using the DeLong method. Association of selected proteins with cognitive decline was investigated using multivariate binary regression models with MMSE change at the last available follow-up visit (<−2 or ≥−2) as dependent variable while entering all selected proteins in the model. We explored the effect of the following confounders: age, sex, baseline MMSE score, time to follow-up, and presence of cerebral AD by entering them into the model before considering protein concentrations. We used a forward selection method based on the significance of the score statistics to avoid overfitting.

To further investigate proteins selected by EN regularization, the proteins were searched in the UniProt database [20] and their entry number was then subsequently used within the Reactome database [21]. This analysis used hypergeometric distribution to determine which pathways and biological reactions were over-represented within the dataset. The false discovery rate (FDR) was further calculated using the Benjamini–Hochberg approach. We only considered pathways with both a *p*-value < 0.05 and FDR < 0.25 as relevant. Over-represented pathways were then manually grouped into broader ontology-based categories (Supplementary Materials Tables S4–S6). Pathways related to coronavirus infection were excluded from the analysis (8 pathways).

## 3. Results

Clinical and demographic characteristics of the cohort are shown in Table 1. CSF AD status, APOE ε4 carrier status, sex, clinical dementia rating (CDR), and Mini Mental State Examination test (MMSE) scores were significantly different between NPS positive and NPS negative groups. There was no significant difference in age between NPS positive and negative individuals. Longitudinal data were available in 69 participants.

To identify proteins differentially expressed between NPS positive and negative groups, we performed a Mann–Whitney U-Test. This approach identified 154 proteins with different CSF concentrations (Supplementary Materials Table S2). The relationship between proteins and total NPI-Q score was additionally measured with correlation coefficients and 24 proteins were identified (Supplementary Materials Table S3).

**Figure 1:**
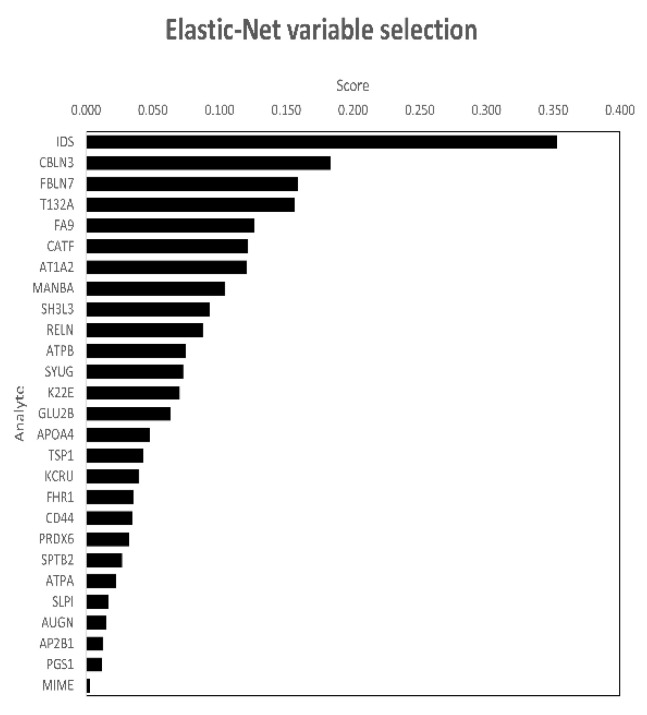
EN variable selection and protein scores for total NPI positive individuals.Proteins sorted by absolute value of EN regression score (*x*-axis); IDS-Iduronate 2-sulfatase; CBLN3- Cerebellin-3; FBLN7-Fibulin-7; T132A-Transmembrane protein 132A; F9-Coagulation factor IX; CTSF-Cathepsin F; ATP1A2-Sodium/potassium-transporting ATPase subunit alpha-2; MANBA-Beta-mannosidase; SH3L3-SH3 domain-binding glutamic acid-rich-like protein 3; RELN-reelin; ATPB-ATP synthase subunit beta, mitochondrial; SYUG-Gamma-synuclein; K22E-Keratin, type II cytoskeletal 2 epidermal; GLU2B-Glucosidase 2 subunit beta; APOA4-Apolipoprotein A-IV; TSP1-Thrombospondin-1; KCRU-Creatine kinase U-type, mitochondrial; FHR1-Complement factor H-related protein 1; CD44-CD44 antigen; PRDX6-Peroxiredoxin-6; SPTB2-Spectrin beta chain, non-erythrocytic 1; ATPA; ATP synthase subunit alpha, mitochondrial; SLPI-Antileukoproteinase; AUGUN-Augurin; AP2B1-AP-2 complex subunit beta; PGS1-CDP-diacylglycerol-glycerol-3-phosphate 3-phosphatidyltransferase, mitochondrial; and MIME-Mimecan.

**Figure 2:**
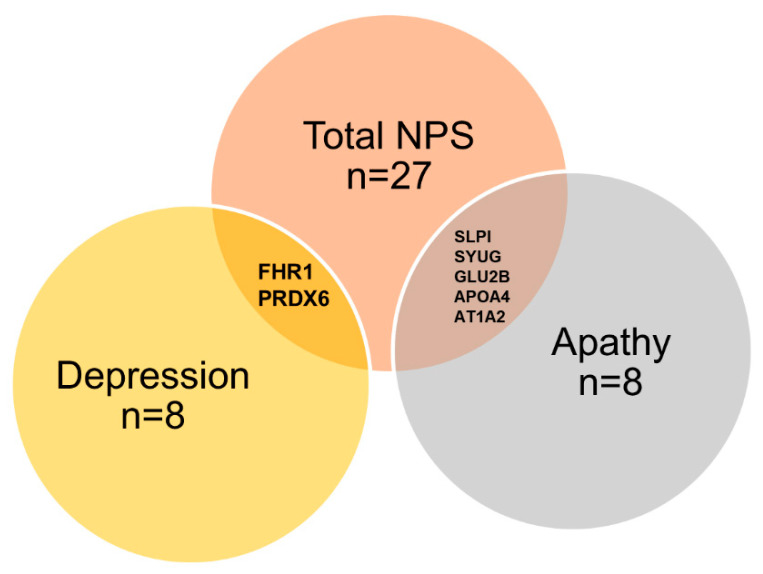
Venn diagram of analytes associated with total NPS, depression, or apathy, obtained by EN regression models.

To better identify proteins associated with the occurrence of NPS, we then applied EN regression. This approach selected 27 proteins associated with the presence of NPS (Figure 1, Table 2) out of 790 total proteins (Supplementary Materials Table S1). The proteins selected by EN were identical for the models with and without AD as a variable. We next focused on analyzing the most frequent and persistent NPS during the disease course: depression, apathy, and anxiety. EN selected proteins for depression and dysphoria (Table 3), and apathy and indifference (Table 4) The proteins selected by EN, overlapping between NPS, apathy, and depression, are illustrated as a Venn diagram (Figure 2). Although anxiety was the most prevalent symptom in our cohort (29%; N = 25/87 participants), this approach did not select any proteins related to it.

The Venn diagram is based on the 27 proteins selected by EN. Number of proteins identified as well as the names of those shared between syndromes is shown. The full list of associated proteins is presented in Table 2, Table 3 and Table 4.

**Figure 3:**
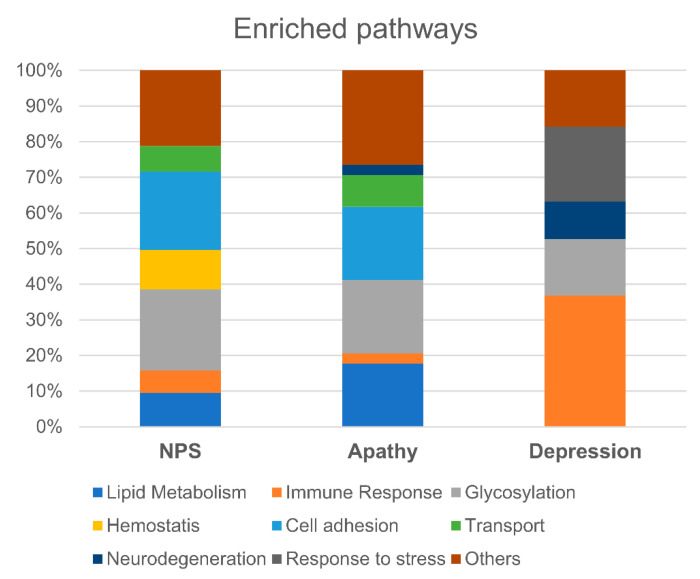
Comparison of enriched pathways between total NPS, depression, and apathy. Above is the pathway enrichment analysis of identified proteins for total NPS, apathy, or depression. The number of over-represented categories within each symptom (expressed as a percentage) is illustrated.

In order to characterize alterations in underlying molecular pathways for the 27 proteins selected with EN for total NPS, depression, and apathy, we used the Reactome database and coarse-grain ontological categories (See Methods and Supplementary Materials Tables S4–S6). The analysis showed overrepresentation of the glycosylation (23%), cell adhesion (22%), haemostasis (11%), lipid metabolism (9%), transport (7%), and immune response (6%) pathways in individuals with a positive NPI-Q score (Figure 3). The category “other pathways” (21%) consisted mainly of transcriptional, regulatory, and carbohydrate metabolism processes. An analogical approach applied to the depression and apathy domains revealed a different proportion of enriched pathways for each domain (Figure 3, Supplementary Material Tables S4–S6). Depression showed a strong neuroinflammatory profile (36% enriched pathways), whereas in apathy cell adhesion and signal transduction pathways (20%) and lipid metabolism pathways (17%) prevailed. Pathways related to glycosylation and protein posttranslational modifications were enriched for NPI-total score, depression, and apathy (Figure 3 and Supplementary Materials Tables S4–S6).

**Figure 4:**
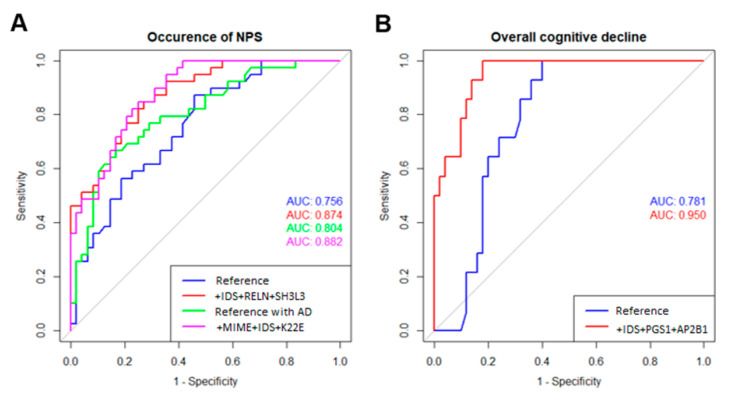
ROC curve comparison for the reference and selected diagnostic models. (**A**) ROC curves and AUCs for the reference models (sex + age + MMSE score) with (green) or without considering cerebral AD (blue) and the final diagnostic models of the occurrence of NPS obtained after addition of three proteins (IDS + RELN + SH3L3) without AD (red) and three proteins (MIME + IDS + K22E) with AD (magenta). (**B**) ROC curves and AUCs for the reference model (age + sex+ baseline MMSE score + time to follow-up + cerebral AD status (blue)) and the final diagnostic model of decline in global cognition (MMSE change at last follow-up <−2) after addition of three proteins (IDS + PGS1 + AP2B1).

We next constructed a minimal diagnostic model for the prediction of NPS using proteomic data for the 27 proteins selected with EN. Our iterative approach reached an optimal diagnostic model with three proteins (i.e., IDS + RELN + SH3L3-AD; selected model without AD; for protein name abbreviations see Figure 1), that together improved the AUC of the ROC curve when compared to the reference model without AD (Figure 4, *p*-value = 0.0008). In addition, sensitivity was improved from the reference model without AD (0.48 to 0.69) whereas specificity was similar (0.81 vs. 0.85). When considering the presence of cerebral AD, our optimal diagnostic model again selected three proteins (i.e., MIME + IDS + K22E + AD; selected model with AD; for protein name abbreviations see Figure 1), that together improved the AUC of the ROC curve when compared to the reference model with AD (Figure 4, *p*-value = 0.033). Sensitivity was higher than for the selected model without AD and adding three proteins (selected model with AD) improved it further (0.64 to 0.74). Specificity was similar for both models (0.83 vs. 0.81). In addition, three proteins (i.e., IDS + FHR1 + PGS1; for protein name abbreviations see Figure 1) were associated with decline in global cognition at the last follow-up visit (42 months from baseline on average; Table 5).

## 4. Discussion

Using a state-of-the-art proteomic approach, we identified 27 proteins associated with NPS. This selection based on EN regression appeared to be independent of the presence of the core AD pathology, suggesting distinct pathophysiological processes are at play in NPS. Exploratory analysis identified specific protein profiles and pathway alterations related to apathy and depression. Furthermore, three out of the 27 selected proteins were associated with cognitive decline at follow-up.

Among the proteins different between groups, none was previously reported in association with NPS. The levels of Apolipoprotein E and two other members of the apolipoprotein family APOA4 and APOH differed between groups with and without NPS. These proteins have been linked to AD and cognitive impairment [22], and carrying the APOEe4 allele has been related to NPS in AD previously [23]. A further selected protein is CBPN, which, although not known to be associated with NPS, has been identified as a risk factor of developing post-operative delirium [24].

To identify proteins independently associated to NPS, we applied a regression approach (EN), which selected a panel of 27 proteins. Of note, despite our relatively small sample size and large panel of proteins, these results are not driven by type 1 errors, as demonstrated by increasing the confidence interval to 99%, which produced the same results. A total of seven proteins were consistently selected by all used approaches: APOA4, SLPI, MIME, FBLN7, SYUG, CATF, and PGS1. These proteins relate to neuroinflammation, adhesion, transportation, oxidation, haemostasis, and synaptic plasticity processes. To the best of our knowledge, none of these proteins have been previously associated with NPS. Of note, the exact same set of 27 proteins were selected by EN when CSF AD status was considered in the models for the prediction of NPS. This indicates that the presence of cerebral AD pathology per se does not have an influence on the associations between the identified proteins and NPS, and suggests that at the proteome level the pathological changes underlying NPS are different and largely independent from the core AD pathology. Of note, imaging studies indicated that NPS and AD share only some selected alterations of neural circuits [25] and show a different functional connectivity [26].

When exploring associations with single neuropsychiatric syndromes, we found distinct molecular and pathway enrichment profiles for apathy and depression, suggesting that specific pathological processes may underlie the different NPS domains Similarly, recent studies report distinct brain circuits and functional connectivities related to the development of single neuropsychiatric syndromes [26,27], further supporting the hypothesis of distinct pathophysiological processes, at both connectivity and molecular levels [7].

Only a few studies investigated a priori chosen proteins, such as sICAM-1, IL-10, CRP, and reported associations with NPS in general as well as with apathy and depression [28,29]. Such results are difficult to compare with ours due to the differences in methodology applied (in particular, targeted molecule selection vs. untargeted omics approaches). In our study, we did not identify any proteins associated with anxiety. We hypothesize that other biological (i.e., not at proteome level) and environmental aspects contribute to the pathogenesis of anxiety. The important role of environmental factors in anxiety has been previously highlighted by others. Indeed, individuals with unmet psychological needs, especially in daytime activities, psychological distress, memory and communication impairment, stressful life events, and dependency are more vulnerable to anxiety [30,31].

Inflammatory processes have been linked to several NPS domains, especially to depression. Major depression is linked to neuroinflammatory processes [32,33] that may be a consequence of microglia activation and of neuroinflammatory markers crossing through the blood–brain barrier [33]. Moreover, a specific inflammatory etiology for late-onset depression in older adults has been suggested. CRP was reported to be a marker of depression with a predictive value of progression to dementia [34,35]. Several proteins, among them Insulin-like Growth Factor-1 (IGF-1), Metalloproteinase type 1 (TIMP-1), and Vascular Cell Adhesion Molecule type 1 (VCAM-1) were reported to mediate association between dementia and depression [36].

In our study, two proteins involved in complement activation, namely FHR1 and C1RL, were selected by EN for the individuals with depression and dysphoria. FHR 1 belongs to the Factor H (FH) protein family. High levels of FH, a member of this protein family, were associated with geriatric depression, suggesting that the alternative pathway of the complement contributes to the development of geriatric depression [37].

Interestingly, both for NPS in general as well as for depression and apathy, there was an enrichment in pathways related to glycosylation and protein posttranslational modification, which could affect protein trafficking for instance. Dysfunction in glycosylation has been reported in rheumatoid arthritis [38] and in cancer [39], but also in several neuropsychiatric diseases, such as post-traumatic stress disorder, depression, and schizophrenia [40,41,42]. In AD dementia and pre-dementia stages, an increase in glycans in CSF has been observed [43,44]. Because impairment in glycosylation takes place in early AD stages, glycans are considered an interesting diagnostic and therapeutic target, and could be modulated pharmacologically [44].

To explore whether a protein-based biomarker diagnostic tool may be useful to detect pathophysiological alterations underlying NPS, we determined two optimal diagnostic models including three proteins each: one without AD pathology status (IDS + RELN + SH3L2) and the second considering AD status (MIME + IDS + K22E + AD). The model with AD was more sensitive, as was expected, because AD status may be a predictive factor for NPS. The proteins most relevant for predicting NPS are different in the context of AD, suggesting that some proteins associated with NPS interact with AD pathology. To the best of our knowledge, none of these proteins have previously been associated with NPS. MIME and IDS have been linked to AD [45,46]. A differential expression of SH3L3 has been identified in a single study of both sporadic AD and rapid progressive AD patients compared to controls [47]. RELN is involved in the APOE biochemical pathway and inhibits regulators of tau phosphorylation [48,49].

We further found that three proteins, i.e., IDS, FHR1, and PGS1, were independently associated with faster global cognitive decline even after considering the presence of AD in the models. These findings suggest that some protein alterations related to NPS indicate pathological processes that may contribute to faster cognitive decline, in addition to the effects of AD pathology, and may explain the previously observed association of NPS with more rapid cognitive decline.

Our work represents the first study applying an unbiased proteomic approach to investigate NPS in older people with cognitive decline. We report CSF proteome alterations related to NPS in general, and depression and apathy in particular, and address relationships of these alterations with biologically defined AD and with cognitive decline at follow-up. However, several limitations should be acknowledged. First, we focused on the most common and persistent single NPS domains and did not address other syndromes. Due to the relatively small cohort size, some other NPS domains have been not frequent enough to be properly considered in the analysis. We included only community-dwelling subjects with normal cognition, MCI, and mild dementia, investigated in a memory clinic setting, while excluding individuals with more severe dementia or major psychiatric disorders and severe syndromes that may interfere with cognition. Therefore, our results are not fully representative for elderly people in general. Although the subgroups of participants with and without NPS were well balanced in terms of age and sex, there was an overrepresentation of AD patients in the NPS cohort, given that NPS often accompany developing AD. Accordingly, our results should be considered as preliminary and need to be validated in larger and independent cohorts.

## 5. Conclusions

Our study demonstrates the value of proteome profiling to uncover pathway alterations associated with NPS. An important finding is that the pathophysiological processes underlying NPS appear to be at least partially distinct from AD pathology. Furthermore, these proteome and pathways alterations are related to, and may accelerate clinical disease progression. The identification of distinct molecular endophenotypes of NPS could be useful to develop targeted treatment to both reduce NPS and slow cognitive decline in older people.

## Figures and Tables

**Table 1:** Clinical and biomarker characteristics of NPS negative and positive subjects.

Clinical Characteristics	NPI Negative	NPI Positive	*p*-Value
Mean Score ± SD	N = 48	N = 39	
Age years	68.19 ± 8.01	71.82 ± 6.1	0.065
Sex female N (%)	34 (70.8)	24 (61.5)	0.02
Cognitive impairment N (%)	17 (35.4)	28 (71.8)	0.001
APOE ε4 carrier status N (%)	8 (16.7)	21 (53.8)	0.002
CSF AD status N (%)	7 (14.6)	23 (59)	0.004
MMSE	28.4 ± 1.92	26.03 ± 3.44	0
Depression N (% with AD)		8 (47.1)	
Anxiety N (% with AD)		16 (59.3)	
Apathy N (% with AD)		13 (68.2)	

Legend: N—absolute number of individuals.

**Table 2:** EN variable selection for NPS positive individuals.

Protein Name	Gene Name	Protein Full Name	Protein ID	VIF
AUGN	ECRG4	Augurin	Q9H1Z8	1.8
IDS	IDS	Iduronate 2-sulfatase	P22304	1.93
FHR1	CFHR1	Complement factor H-related protein 1	Q03591	1.97
TSP1	THBS1	Thrombospondin-1	P07996	2.08
K22E	KRT2	Keratin, type II cytoskeletal 2 epidermal	P35908	2.15
SPTB2	SPTBN1	Spectrin beta chain, non-erythrocytic 1	Q01082	2.46
AP2B1	AP2B1	AP-2 complex subunit beta	P63010	2.51
SH3L3	SH3BGRL3	SH3 domain-binding glutamic acid-rich-like protein 3	Q9H299	2.51
CD44	CD44	CD44 antigen	P16070	2.82
PGS1	PGS1	CDP-diacylglycerol-glycerol-3-phosphate 3-phosphatidyltransferase, mitochondrial	Q32NB8	2.87
FA9	F9	Coagulation factor IX	P00740	3.09
APOA4	APOA4	Apolipoprotein A-IV	P06727	3.37
SLPI	SLPI	Antileukoproteinase	P03973	3.67
CBLN3	CBLN3	Cerebellin-3	Q6UW01	3.97
MANBA	MANBA	Beta-mannosidase	O00462	4.14
AT1A2	ATP1A2	Sodium/potassium-transporting ATPase subunit alpha-2	P50993	4.66
PRDX6	PRDX6	Peroxiredoxin-6	P30041	5.4
RELN	RELN	Reelin	P78509	5.64
MIME	OGN	Mimecan	P20774	7.26
T132A	T132A	Transmembrane protein 132A	Q24JP5	8.24
FBLN7	FBLN7	Fibulin-7	Q53RD9	13.75
SYUG	SNCG	Gamma-synuclein	O76070	14.49
GLU2B	PRKCSH	Glucosidase 2 subunit beta	P14314	19.37
CATF	CTSF	Cathepsin F	Q9UBX1	24.65
ATPA	ATP5F1A	ATP synthase subunit alpha, mitochondrial	P25705	168.89
ATPB	ATP5F1B	ATP synthase subunit beta, mitochondrial	P54709	2131.14
KCRU	CKMT1A	Creatine kinase U-type, mitochondrial	P12532	2547.26

Legend: VIF-variance inflation factor.

**Table 3:** EN variable selection for individuals positive for depression and dysphoria domain.

Protein Name	Gene Name	Protein Full Name	Protein ID
GLT10	GALNT10	Polypeptide N-acetylgalactosaminyltransferase 10	Q86SR1
FHR1	CFHR1	Complement factor H-related protein 1	Q03591
CASC4	GOLM2	Protein GOLM2	Q6P4E1
CNTFR	CNTFR	Ciliary neurotrophic factor receptor subunit alpha	P26992
PRDX6	PRDX6	Peroxiredoxin-6	P30041
PRDX2	PRDX2	Peroxiredoxin-2	P32119
OX2G	CD200	OX-2 membrane glycoprotein	P41217
C1RL	C1RL	Complement C1r subcomponent-like protein	Q9NZP8

**Table 4:** EN variable selection for individuals positive for apathy and indifference domain.

Protein Name	Gene Name	Protein Full Name	Protein ID
SLPI	SLPI	Antileukoproteinase	P03973
UBQL2	UBQLN2	Ubiquilin-2	Q9UHD9
CADM2	CADM2	Cell adhesion molecule 2	Q8N3J6
CD048	C4orf48	Neuropeptide-like protein C4orf48	Q5BLP8
SYUG	SNCG	Gamma-synuclein	O76070
GLU2B	PRKCSH	Glucosidase 2 subunit beta	P14314
APOA4	APOA4	Apolipoprotein A-IV	P06727
AT1A2	ATP1A2	Sodium/potassium-transporting ATPase subunit α2	P50993

**Table 5:** Associations between selected proteins and MMSE change at last follow-up (<−2 or ≥−2 point). Standardized β-coefficients and *p*-value for selected proteins and confounders are shown.

MMSE Decline at Last Follow-Up		
Variable	Coeff.	*p*-Value
Age	1.061	0.455
Sex	12.947	0.033
Baseline Score	1.083	0.621
Time to follow-up	1.046	0.186
**CSF AD status**	**7.340**	**0.049**
**IDS**	**0.193**	**0.006**
**FHR1**	**6.853**	**0.002**
**PGS1**	**2.508**	**0.045**

## Supplementary Materials

The following supporting information can be downloaded at: https://www.mdpi.com/article/10.3390/cells11061030/s1, Table S1: A total of 790 proteins investigated in this study; Table S2: Proteins differentially expressed between NPS positive and negative groups; Table S3: Association between total NPI-Q > 0 and analyte; Table S4: Ontological categories for total NPI-Q; Table S5: Ontological categories for depression; Table S6: Ontological categories for apathy; Table S7: Proteins differentially expressed in participants with or without NPS in participants without AD (left) or presenting cerebral AD (right); Table S8: Mean, median, and range for the NPI-Q total score and for depression, apathy, and anxiety domains.
