# Supplementary material for: Cerebrospinal Fluid Proteome Alterations Associated with Neuropsychiatric Symptoms in Cognitive Decline and Alzheimer’s Disease
Source: Cells. 2022 Mar 18;11(6):1030. doi: 10.3390/cells11061030 (PMC8947516; doi:10.3390/cells11061030)
Supplement: Supplementary file 1 [file cells-11-01030-s001.zip › cells-1629435-supplementary.pdf]

Supplementary methods:

An exploratory analysis approach on subgroups within the cohort was performed in which participants were classified according to both the occurrence of NPS (NPI-Q > 0) and the presence of cerebral AD pathology (CSF p-tau181/Aβ1–42 ratio > 0.0779). Given the small sample size of NP-/AD+ participants we could not fit neither a binomial regression nor an Elastic model on the available data. Both models were greatly overfit which was expected considering how the number of variables surpasses the number of samples. As a result, we explored the data with Mann–Whitney U test looking at the concentration of CSF proteins between with or without NPS in AD- participants or in AD+ participants. These tests did not uncover any further differentially expressed proteins but only identified a subset of the proteins that were different between groups as shown below (**Supplementary Materials Table S7**). These results are difficult to interpret since only 15 participants presenting NPS are included in the AD- group and in the AD+ only 7 participants do not have NPS. This suggests that while there is some interaction between AD and NPS, the proteins identified in association with NPS are largely independent of the presence of AD, further confirming results observed in the whole cohort in models considering AD status as a covariable.

790 proteins investigated in this study

A1AT	C1R	CNTN2	ECM1	GLT10	K2C6AP1	MUC18	PCP4	RCN2	SYT7
A1BG	C1RL	CNTN4	ECM2	GLT13	KAIN	MXRA8	PCSK1	RELN	SYUB
A2AP	C1S	CNTP2	EDN3	GLT15	KCRM	MYG	PCSK5	RET4	SYUG
A2GL	C99L2	CNTP4	EFC14	GLT18	KCRU	MYH15	PDGFB	RGMA	SZT2
A4	CA2D1	CO1A1	EFNA1	GLU2B	KIT	MYH7	PDIA1	RGMB	T132A
AACT	CAB45	CO1A2	EFNB2	GMFB	KLK6	MYOC	PDIA3	RNAS1	T132D
AATC	CAD10	CO2	EH1L1	GNAS3	KLKB1	NAC2	PDIA6	RNAS4	TAGL
ABCA2	CAD11	CO3	EMIL1	GNPTG	KNG1	NAR3	PDYN	RNAS6	TAGL2
ABHEA	CAD13	CO3A1	EMIL2	GOLI4	KPYM	NBL1	PEBP1	RNT2	TAGL3
ACBP	CAD15	CO4A	ENDD1	GOLM1	L1CAM	NCAM1	PEBP4	ROBO1	TBD2A
ACON	CAD20	CO4B	ENOA	GP158	LAC2	NCAM2	PEDF	ROR1	TCO2
ACTAP2	CADH2	CO5	ENOG	GPC1	LAIR1	NCAN	PENK	RTN4R	TEFF1
ACTB	CADH4	CO6	ENPL	GPR37	LAMA2	NCHL1	PGAM1	S10A6	TENR
ACYP2	CADH5	CO6A1	ENPP2	GPX3	LAMB2	NDRG2	PGBM	S10A7	TENX
ADA10	CADH6	CO6A3	EPDR1	GRIA4	LAMC1	NEC1	PGCB	S10A8	TETN
ADA11	CADH7	CO7	EPHA4	GRP78	LAMP1	NEC2	PGK1	S10A9	TGBR3
ADA22	CADH8	CO8A	EPHA5	GSLG1	LAMP2	NEDD8	PGRC1	S38AA	TGON2
ADA23	CADM1	CO8B	EPHA7	GSTP1	LBP	NEGR1	PGRP2	S39AA	THBG
ADAM9	CADM2	CO8G	EPHAA	GUAD	LCAT	NELL2	PGS1	SAMP	THIO
ADIRF	CADM3	CO9	EPHB2	H14	LCN1	NENF	PGS2	SAP	THRB
ADT2	CADM4	COCH	EPHB6	H6ST3	LDHB	NEO1	PHLD	SAP3	THSD4
AEBP1	CAH1	COF1	ERP44	HABP2	LEG1	NEU1	PI16	SBP1	THY1
AFAM	CAH11	COFA1	ESAM	HBA	LFG2	NEUG	PIANP	SCG1	TICN1
AGRIN	CAH2	COIA1	ETBR2	HBB	LFNG	NEUM	PIMT	SCG2	TICN2
AKA11	CAH3	COL12	EXTL2	HBD	LG3BP	NEUS	PITH1	SCG3	TICN3
ALBU	CALB1	COMP	F13B	HEG1	LIGO1	NFASC	PKHB1	SCN3B	TIMP1
ALDOA	CALB2	COTL1	FA10	HELZ	LMAN1	NFH	PLD3	SCN4B	TIMP2
ALDOC	CALM	CP089	FA12	HEMO	LMAN2	NFM	PLD4	SCRG1	TITIN
ALS	CALR	CPN2	FA5	HEP2	LPHN1	NID1	PLMN	SE6L1	TM59L
AMBP	CANT1	CPVL	FA69C	HEXA	LPHN3	NID2	PLOD1	SE6L2	TMED4
AMD	CART	CRAC1	FA9	HGFA	LRC4B	NIN	PLSL	SELM	TMG1
ANFC	CASC4	CRDL1	FABP4	HGFL	LRC57	NOE1	PLTP	SEM3B	TNR21
ANGI	CATB	CREL1	FABP5	HRG	LRCH3	NOV	PLXB2	SEM3G	TP53B
ANGL7	CATD	CRHBP	FABPH	HS71L	LRP1	NPC2	PMGT1	SEM4B	TPIS
ANGT	CATF	CRLD1	FAM3C	HSP13	LRP11	NPDC1	PNOC	SEM6A	TPM1
ANT3	CATH	CRP	FAT2	HSP7C	LRP2	NPTX1	PON1	SEM6D	TPP1
ANXA1	CATL1	CS010	FBLN1	HTRA1	LRP8	NPTX2	POSTN	SEM7A	TRFE
AP2B1	CATS	CSF1	FBLN3	HYOU1	LSAMP	NPTXR	PPGB	SEP_15	TRY1
APC	CATZ	CSF1R	FBLN5	IBP2	LSR	NPY	PPIA	SEPP1	TSP1
APLP1	CBG	CSMT1	FBLN7	IBP3	LTBP1	NRCAM	PPIB	SETX	TSP2
APLP2	CBLN1	CSPG2	FBN1	IBP4	LTBP2	NRN1	PPIC	SEZ6	TSP4
APOA2	CBLN3	CSTN1	FCG2A	IBP5	LTBP4	NRP1	PPN	SFRP3	TTHY
APOA4	CBPB2	CSTN2	FCGBP	IBP6	LUM	NRP2	PRAP1	SFRP4	TYB10
APOD	CBPE	CSTN3	FETUA	IBP7	LUZP2	NRX1A	PRC2C	SGCE	TYB4
APOE	CBPN	CTGF	FETUB	IC1	LY6H	NRX2A	PRDX1	SH3L1	UB2L3
APOH	CBPQ	CUTA	FGD6	ICAM2	LYAG	NRX3A	PRDX2	SH3L3	UBBP1
ARHG5	CCKN	CXCL7	FGFR1	ICAM5	LYSC	NSG1	PRDX6	SHBG	UBQL2
ARSA	CCL2	CXL16	FGFR2	ICOSL	LYST	NTNG1	PRELP	SHPS1	UCHL1
ASAH1	CD048	CYC	FGFR3	IDD	LYVE1	NTRI	PRG4	SHSA5	UFO
ASPG	CD109	CYTA	FHL1	IDS	MA1A1	NTRK2	PRIO	SHSA6	UK114
ASPH	CD14	CYTB	FHR1	IGF2	MA1C1	NUCB1	PRKDC	SIAE	VAMP2
AT1A2	CD166	CYTC	FHR2	IGHG1	MA2A1	NUCB2	PROC	SIAT2	VAS1
AT1A3	CD44	CYTL1	FIBA	IGKC	MA2A2	NXPH1	PROF1	SKP1	VASN
ATPA	CD59	DAF	FIBB	IGLO5	MAG	NXPH4	PROS	SLIK1	VCAM1
ATPB	CDC27	DAG1	FINC	IGS21	MANBA	OAF	PRRT2	SLIK4	VGF
ATRN	CELR2	DCC	FMOD	IGSF1	MANS1	ODP2	PRRT3	SLPI	VIME
ATS1	CERU	DCD	FOLR2	IGSF8	MARCS	ODPB	PRUN2	SMOC1	VSIG4
ATS2	CFAB	DDAH1	FRIL	IL6RB	MASP1	OLFL3	PRVA	SMS	VTDB
ATS3	CFAD	DDR1	FRS1L	IMPA3	MDHC	OMD	PTGDS	SNED1	VTM2A
ATS8	CFAH	DHE3	FSIP2	IPSP	MDHM	OMGP	PTHD2	SODC	VTM2B
AUGN	CFAI	DHPR	FSTL1	ISK6	MEG10	OPCM	PTN	SODE	VTNC
B2MG	CGRE1	DIA1R	FSTL4	ISLR	MEGF8	OSTP	PTPR2	SORC1	VWA1
B3GN1	CH10	DIAC	FSTL5	ISLR2	METRL	OX2G	PTPRD	SORC2	VWC2
B3GN2	CH3L1	DIDO1	FUCO	ITIH1	MFAP4	P3IP1	PTPRF	SORC3	VWF
B4GN1	CH60	DKK3	FUCO2	ITIH2	MFSD6	PAG15	PTPRG	SORT	WFDC1
B4GT1	CHAD	DMP4	G3P	ITIH3	MIA	PALM	PTPRK	SPB6	WFDC2

BAI1	CHRD	DNER	G6PI	ITIH4	MIA3	PARK7	PTPRN	SPD2B	WFKN2
BAI2	CHSTA	DNJA1	GALT2	ITIH5	MICU1	PCD16	PTPRS	SPIT2	WSCD1
BAI3	CI117	DNJC3	GALT6	ITM2B	MIME	PCD17	PTPRT	SPON1	X1433B
BASI	CL049	DNS2A	GALT7	JAM2	MLEC	PCDC2	PTPRZ	SPRC	X1433Z
BASP1	CLC11	DPP10	GANAB	JAM3	MMP2	PCDGK	PUR2	SPRL1	X1A68
BGH3	CLN5	DPP2	GAS6	K0319	MMRN2	PCDGM	PVR	SPTB2	X1C12P1
BLVRB	CLUS	DPP6	GDF11	K154L	MOES	PCDH1	PVRL1	SPTN5	X4F2
BMP1	CMGA	DSC2	GDN	K1C10	MOG	PCDH7	PXDC2	STC2	X7B2
BTD	CNDP1	DSC3	GELS	K1C14	MROH7	PCDH8	QSOX1	STML2	XYLT1
C163A	CNPY4	DSG2	GFRA2	K1C9	MSLN	PCDH9	QSOX2	STMN1	YIPF3
C1QB	CNR1	DYH10	GGH	K22E	MT1E	PCNT	R4RL2	STX12	ZA2G
C1QC	CNTFR	DYH5	GLDN	K2C1	MT3	PCOC1	RARR2	SUCO	ZP2
C1QT4	CNTN1	DYL2	GLRX1	K2C5	MTPN	PCP	RCN1	SUSD5	ZPI

Supplementary Materials Table S1. A total of 790 proteins investigated in this study.

Protein	Mean Difference	p-Value	Protein	Mean Difference	p-Value	Protein	Mean Difference	p-Value
A1BG	-2.269	0.023	DSC3	-1.989	0.047	MIME	-2.305	0.021
A2AP	-2.603	0.009	DYL2	-1.989	0.047	MYH15	-2.865	0.004
ADA10	-2.257	0.024	ECM2	-2.233	0.026	MYH7	-3.317	0.001
ADIRF	-2.103	0.035	EFNA1	-2.151	0.031	NDRG2	-2.370	0.018
AEBP1	-2.910	0.004	ENOA	-2.187	0.029	NEDD8	-2.612	0.009
AFAM	-2.007	0.045	ENPL	-2.160	0.031	OAF	-2.612	0.009
ALDOA	-2.115	0.034	F13B	-3.100	0.002	OLFL3	-2.151	0.031
AMBP	-3.380	0.001	FA10	-2.898	0.004	OMD	-1.979	0.048
ANGI	-3.281	0.001	FABPH	-3.010	0.003	OSTP	-2.007	0.045
ANT3	-2.287	0.022	FBLN1	-2.124	0.034	PARK7	-2.567	0.010
APOA2	-2.278	0.023	FBLN3	-2.260	0.024	PCOC1	-2.368	0.018
APOA4	-2.892	0.004	FBLN7	-2.269	0.023	PGBM	-2.341	0.019
APOE	-2.820	0.005	FETUA	-2.011	0.044	PGK1	-1.961	0.050
APOH	-2.712	0.007	FETUB	-3.263	0.001	PGRP2	-2.956	0.003
BGH3	-2.124	0.034	FIBA	-2.395	0.017	PGS1	-2.123	0.034
BMP1	-2.296	0.022	GELS	-2.404	0.016	PLMN	-2.820	0.005
C163A	-1.961	0.050	GLDN	-3.100	0.002	PON1	-2.540	0.011
C1RL	-2.504	0.012	GMFB	-2.233	0.026	PPIA	-2.187	0.029
CAD11	-2.233	0.026	GNPTG	-2.007	0.045	PROC	-2.317	0.020
CAD20	-2.185	0.029	HABP2	-2.847	0.004	RARR2	-2.368	0.018
CADH5	-2.793	0.005	HEG1	-2.513	0.012	RET4	-3.742	0.000
CADM4	-1.961	0.050	HELZ	-2.621	0.009	RNAS4	-2.404	0.016
CALB2	-2.892	0.004	HEMO	-2.350	0.019	RNAS6	-2.197	0.028
CART	-1.995	0.046	HEP2	-2.802	0.005	SAMP	-2.052	0.040
CATF	-2.196	0.028	HRG	-2.838	0.005	SEP15	-2.106	0.035
CATH	-2.847	0.004	HSP7C	-2.169	0.030	SH3L1	-2.142	0.032
CATS	-1.961	0.050	IBP3	-2.531	0.011	SLPI	-2.721	0.007
CBG	-2.115	0.034	IBP4	-2.151	0.031	SMOC1	-2.829	0.005
CBPB2	-2.061	0.039	IBP5	-2.133	0.033	SNED1	-1.981	0.048
CBPN	-2.992	0.003	IBP6	-2.214	0.027	SODE	-2.178	0.029
CCL2	-1.998	0.046	IBP7	-2.214	0.027	SPON1	-2.016	0.044
CD14	-2.431	0.015	IC1	-3.155	0.002	STMN1	-2.341	0.019
CFAD	-2.910	0.004	IDD	-2.255	0.024	SYUG	-2.368	0.018

CFAH	-3.010	0.003	IPSP	-2.838	0.005	SZT2	-3,028	0.002
CFAI	-2.151	0.031	ITIH2	-2.531	0.011	TAGL3	-2,436	0.015
CH3L1	-2.585	0.010	ITIH4	-2.431	0.015	TBD2A	-2,874	0.004
CL049	-2.513	0.012	KAIN	-2.766	0.006	THBG	-2,165	0.030
CLUS	-2.377	0.017	KLKB1	-2.412	0.016	TICN3	-2,187	0.029
CO2	-2.133	0.033	KNG1	-2.766	0.006	TIMP2	-2,124	0.034
CO3	-2.468	0.014	KPYM	-2.269	0.023	TMED4	-2,250	0.024
CO5	-2.495	0.013	LAMA2	-2.269	0.023	TSP4	-2,863	0.004
CO6A3	-2.332	0.020	LDHB	-2.025	0.043	TYB10	-2,187	0.029
CO7	-2.522	0.012	LEG1	-2.341	0.019	VASN	-2,251	0.024
CO8A	-2.612	0.009	LG3BP	-1.975	0.048	VTDB	-2,359	0.018
CO8B	-2.156	0.031	LMAN1	-2.085	0.037	VTNC	-2,965	0.003
CO8G	-2.296	0.022	LTBP2	-2.314	0.021	X1433B	-2,685	0.007
CPVL	-2.139	0.032	LTBP4	-1.989	0.047	X1433Z	-2,603	0.009
CRDL1	-2.212	0.027	LUM	-2.784	0.005	X1C12P1	-2,730	0.006
CRLD1	-2.097	0.036	LYSC	-2.910	0.004	ZA2G	-2,739	0.006
CSF1	-2.142	0.032	MA2A1	-3.204	0.001			
CXL16	-2.033	0.042	MEG10	-1.970	0.049			
CYTB	-2.287	0.022	MIA	-2.617	0.009			

Supplementary Materials Table S2. Proteins differentially expressed between NPS positive and negative groups (Mann–Whitney U test, p-value < 0.005).

Protein	Coeff.	Protein	Coeff.	Protein	Coeff.
DNS2A	-0.3	LRP11	-0.21	MANBA	0.21
CO1A1	-0.26	PCP	-0.21	CLN5	0.24
CNTN1	-0.25	PDYN	-0.21	TSP1	0.24
MOG	-0.25	PCDH7	-0.2	CADM2	0.25
EPHB2	-0.25	ANGI	-0.2	WSCD1	0.25
NPY	-0.23	NEGR1	-0.19	KCRU	0.28
BAI3	-0.23	IDS	-0.19	TPIS	0.28
THRB	-0.22	K1C14	-0.17	KAIN	0.31

Supplementary Materials Table S3. Association between total NPI-Q > 0 and analytes (only significant values showed).

Pathway identifier	Pathway name	p-value	False-discovery rate
	**Lipid Metabolism**		
R-HSA-174824	Plasma lipoprotein assembly, remodeling, and clearance	0.013*	0.097*
R-HSA-8963901	Chylomicron remodeling	0.024*	0.097*
R-HSA-8963888	Chylomicron assembly	0.024*	0.097*
R-HSA-8866427	VLDLR internalisation and degradation	0.028*	0.097*
R-HSA-8964038	LDL clearance	0.045*	0.097*
R-HSA-8963898	Plasma lipoprotein assembly	0.045*	0.097*
R-HSA-8963889	Assembly of active LPL and LIPC lipase complexes	0.045*	0.097*
R-HSA-8964043	Plasma lipoprotein clearance	0.077	0.097*
R-HSA-8963899	Plasma lipoprotein remodeling	0.077	0.097*
R-HSA-975634	Retinoid metabolism and transport	0.101	0.101*
R-HSA-1483257	Phospholipid metabolism	0.404	0.404
R-HSA-556833	Metabolism of lipids	0.846	0.846
	**Immune response**		
R-HSA-182218	Nef Mediated CD8 Down-regulation	0.019*	0.097*
R-HSA-1483148	Synthesis of PG	0.019*	0.097*
R-HSA-167590	Nef Mediated CD4 Down-regulation	0.024*	0.097*
R-HSA-6798695	Neutrophil degranulation	0.027*	0.097**
R-HSA-177504	Retrograde neurotrophin signalling	0.033*	0.097*
R-HSA-2132295	MHC class II antigen presentation	0.046*	0.097*
R-HSA-168249	Innate Immune System	0.061	0.097*
R-HSA-168256	Immune System	0.161	0.161*
R-HSA-877300	Interferon gamma signaling	0.374	0.374
R-HSA-913531	Interferon Signaling	0.508	0.508
R-HSA-1280218	Adaptive Immune System	0.681	0.681
R-HSA-1280215	Cytokine Signaling in Immune system	0.866	0.866
	**Glycosylation and other posttranslational modifications**		
R-HSA-2206296	MPS II - Hunter syndrome	0.002*	0.097*
R-HSA-1630316	Glycosaminoglycan metabolism	0.003*	0.097*
R-HSA-3656244	Defective B4GALT1 causes B4GALT1-CDG (CDG-2d)	0.019*	0.097*
R-HSA-3656243	Defective ST3GAL3 causes MCT12 and EIEE15	0.019*	0.097*
R-HSA-3656225	Defective CHST6 causes MCDC1	0.019*	0.097*

R-HSA-2206281	Mucopolysaccharidoses	0.026*	0.097*
R-HSA-2022857	Keratan sulfate degradation	0.0309*	0.097*
R-HSA-879415	Advanced glycosylation endproduct receptor signaling	0.0309*	0.097*
R-HSA-2024101	CS/DS degradation	0.033*	0.097*
R-HSA-2142845	Hyaluronan metabolism	0.04*	0.097*
R-HSA-3781865	Diseases of glycosylation	0.048*	0.097*
R-HSA-2024096	HS-GAG degradation	0.052	0.097*
R-HSA-2022854	Keratan sulfate biosynthesis	0.065	0.097*
R-HSA-9694548	Maturation of spike protein	0.07	0.097*
R-HSA-1638074	Keratan sulfate/keratin metabolism	0.079	0.097*
R-HSA-532668	N-glycan trimming in the ER and Calnexin/Calreticulin cycle	0.081	0.097*
R-HSA-5083635	Defective B3GALTL causes Peters-plus syndrome (PpS)	0.087	0.097*
R-HSA-5173214	O-glycosylation of TSR domain-containing proteins	0.09	0.097*
R-HSA-3560782	Diseases associated with glycosaminoglycan metabolism	0.094	0.097*
R-HSA-1793185	Chondroitin sulfate/dermatan sulfate metabolism	0.112	0.112*
R-HSA-1638091	Heparan sulfate/heparin (HS-GAG) metabolism	0.125	0.125*
R-HSA-3906995	Diseases associated with O-glycosylation of proteins	0.158	0.158*
R-HSA-446203	Asparagine N-linked glycosylation	0.167	0.167*
R-HSA-5173105	O-linked glycosylation	0.242	0.242*
R-HSA-1483206	Glycerophospholipid biosynthesis	0.269	0.269*
R-HSA-597592	Post-translational protein modification	0.255	0.255*
R-HSA-163841	Gamma carboxylation, hypusine formation and arylsulfatase activation	0.0967	0.097*
R-HSA-9683686	Maturation of spike protein	0.014	0.097*
R-HSA-901042	Calnexin/calreticulin	0.0609	0.097*
	**Hemostasis**		
R-HSA-9673218	Defective F9 secretion	0.002*	0.097*
R-HSA-9673240	Defective gamma-carboxylation of F9	0.005*	0.097*
R-HSA-9672396	Defective cofactor function of FVIIIa variant	0.007*	0.097*
R-HSA-9673202	Defective F9 variant does not activate FX	0.007*	0.097*
R-HSA-9672383	Defective factor IX causes thrombophilia	0.007*	0.097*
R-HSA-140834	Extrinsic Pathway of Fibrin Clot Formation	0.012*	0.097*
R-HSA-9673221	Defective F9 activation	0.014*	0.097*

R-HSA-9662001	Defective factor VIII causes hemophilia A	0.017*	0.097*
R-HSA-9668250	Defective factor IX causes hemophilia B	0.021*	0.097*
R-HSA-9671793	Diseases of hemostasis	0.037*	0.097*
R-HSA-140837	Intrinsic Pathway of Fibrin Clot Formation	0.054	0.097*
R-HSA-140877	Formation of Fibrin Clot (Clotting Cascade)	0.09	0.097*
R-HSA-109582	Hemostasis	0.095	0.097*
R-HSA-186797	Signaling by PDGF	0.135	0.097*
R-HSA-114608	Platelet degranulation	0.267	0.267
R-HSA-76005	Response to elevated platelet cytosolic Ca2+	0.276	0.276
R-HSA-977606	Regulation of Complement cascade	0.279	0.279
R-HSA-166658	Complement cascade	0.2989	0.299
R-HSA-76002	Platelet activation, signaling and aggregation	0.477	0.477
	**Cell adhesion and signal transduction**		
R-HSA-5578775	Ion homeostasis	0.007*	0.097*
R-HSA-936837	Ion transport by P-type ATPases	0.007*	0.097*
R-HSA-8866376	Reelin signalling pathway	0.01*	0.097*
R-HSA-216083	Integrin cell surface interactions	0.018*	0.097*
R-HSA-5140745	WNT5A-dependent internalization of FZD2, FZD5 and ROR2	0.003*	0.097*
R-HSA-373760	L1CAM interactions	0.034*	0.097*
R-HSA-5576891	Cardiac conduction	0.038*	0.097*
R-HSA-9651496	Defects of contact activation system (CAS) and kallikrein/kinin system (KKS)	0.038*	0.097*
R-HSA-9703465	Signaling by FLT3 fusion proteins	0.047*	0.097*
R-HSA-164938	Nef-mediates down modulation of cell surface receptors by recruiting them to clathrin adapters	0.052	0.097*
R-HSA-373753	Nephrin family interactions	0.054	0.097*
R-HSA-210991	Basigin interactions	0.059	0.097*
R-HSA-3000170	Syndecan interactions	0.063	0.097*
R-HSA-9682385	FLT3 signaling in disease	0.07	0.097*
R-HSA-445095	Interaction between L1 and Ankyrins	0.072	0.097*
R-HSA-399721	Glutamate binding, activation of AMPA receptors and synaptic plasticity	0.072	0.097*
R-HSA-983712	Ion channel transport	0.073	0.097*
R-HSA-202733	Cell surface interactions at the vascular wall	0.118	0.118*
R-HSA-9662361	Sensory processing of sound by outer hair cells of the cochlea	0.127	0.127*

R-HSA-3000171	Non-integrin membrane-ECM interactions	0.133	0.133*
R-HSA-375165	NCAM signaling for neurite out-growth	0.144	0.144*
R-HSA-422475	Axon guidance	0.15	0.15*
R-HSA-9662360	Sensory processing of sound by inner hair cells of the cochlea	0.156	0.156*
R-HSA-9675108	Nervous system development	0.165	0.165*
R-HSA-9659379	Sensory processing of sound	0.172	0.172*
R-HSA-2682334	EPH-Ephrin signaling	0.2	0.2*
R-HSA-2187338	Visual phototransduction	0.215	0.215*
R-HSA-187037	Signaling by NTRK1 (TRKA)	0.247	0.247*
R-HSA-1500931	Cell-Cell communication	0.271	0.271
R-HSA-166520	Signaling by NTRKs	0.287	0.287
R-HSA-3858494	Beta-catenin independent WNT signaling	0.295	0.295
R-HSA-9711123	Cellular response to chemical stress	0.324	0.324
R-HSA-9006934	Signaling by Receptor Tyrosine Kinases	0.362	0.362
R-HSA-112314	Neurotransmitter receptors and postsynaptic signal transmission	0.398	0.398
R-HSA-9709957	Sensory Perception	0.436	0.436
R-HSA-112315	Transmission across Chemical Synapses	0.487	0.487
R-HSA-195721	Signaling by WNT	0.519	0.519
R-HSA-5684996	MAPK1/MAPK3 signaling	0.519	0.519
R-HSA-5683057	MAPK family signaling cascades	0.563	0.563
R-HSA-5663202	Diseases of signal transduction by growth factor receptors and second messengers	0.641	0.641
R-HSA-2262752	Cellular responses to stress	0.805	0.805
R-HSA-8953897	Cellular responses to external stimuli	0.812	0.812
R-HSA-194315	Signaling by Rho GTPases	0.813	0.813
R-HSA-9716542	Signaling by Rho GTPases, Miro GTPases and RHOBTB3	0.82	0.82
R-HSA-162582	Signal Transduction	0.964	0.964
	**Intra-cellular and cell transport**		
R-HSA-159763	Transport of gamma-carboxylated protein precursors from the endoplasmic reticulum to the Golgi apparatus	0.021*	0.097*
R-HSA-159854	Gamma-carboxylation, Transport, and amino-terminal cleavage of proteins	0.026*	0.097*
R-HSA-381426	Regulation of Insulin-like Growth Factor (IGF) transport and uptake by Insulin-like Growth Factor Binding Proteins (IGFBPs)	0.036*	0.097*
R-HSA-416993	Trafficking of GluR2-containing AMPA receptors	0.04*	0.097*

R-HSA-399719	Trafficking of AMPA receptors	0.072	0.097*
R-HSA-382551	Transport of small molecules	0.097	0.097*
R-HSA-1268020	Mitochondrial protein import	0.146	0.146*
R-HSA-6807878	COPI-mediated anterograde transport	0.219	0.219*
R-HSA-8856825	Cargo recognition for clathrin-mediated endocytosis	0.225	0.225*
R-HSA-8856828	Clathrin-mediated endocytosis	0.297	0.297
R-HSA-199977	ER to Golgi Anterograde Transport	0.314	0.314
R-HSA-948021	Transport to the Golgi and subsequent modification	0.364	0.364
R-HSA-199991	Membrane Trafficking	0.458	0.458
R-HSA-5653656	Vesicle-mediated transport	0.557	0.557
	**Other**		
R-HSA-71387	Metabolism of carbohydrates	0.005*	0.097*
R-HSA-8853383	Lysosomal oligosaccharide catabolism	0.009*	0.009*
R-HSA-5668914	Diseases of metabolism	0.021*	0.097*
R-HSA-71288	Creatine metabolism	0.024*	0.097*
R-HSA-159782	Removal of aminoterminal propeptides from gamma-carboxylated proteins	0.024*	0.097*
R-HSA-159740	Gamma-carboxylation of protein precursors	0.024*	0.097*
R-HSA-8957275	Post-translational protein phosphorylation	0.024*	0.097*
R-HSA-2160916	Hyaluronan uptake and degradation	0.028*	0.097*
R-HSA-5099900	WNT5A-dependent internalization of FZD4	0.036*	0.097*
R-HSA-1643685	Disease	0.036*	0.097*
R-HSA-163210	Formation of ATP by chemiosmotic coupling	0.042*	0.097*
R-HSA-8949613	Cristae formation	0.072	0.097*
R-HSA-9013424	RHOV GTPase cycle	0.077	0.097*
R-HSA-9683701	Translation of structural proteins	0.077	0.097*
R-HSA-9013420	RHOU GTPase cycle	0.079	0.097*
R-HSA-5663084	Diseases of carbohydrate metabolism	0.079	0.097*
R-HSA-397014	Muscle contraction	0.081	0.097**
R-HSA-3299685	Detoxification of Reactive Oxygen Species	0.09	0.097*
R-HSA-6806667	Metabolism of fat-soluble vitamins	0.11	0.11*
R-HSA-437239	Recycling pathway of L1	0.112	0.112*
R-HSA-9694635	Translation of structural proteins	0.112	0.112*
R-HSA-3928665	EPH-ephrin mediated repulsion of cells	0.116	0.116*
R-HSA-1430728	Metabolism	0.132	0.132*

R-HSA-8936459	RUNX1 regulates genes involved in megakaryocyte differentiation and platelet function	0.148	0.148*
R-HSA-1474244	Extracellular matrix organization	0.164	0.164*
R-HSA-977225	Amyloid fiber formation	0.178	0.178*
R-HSA-4086400	PCP/CE pathway	0.2	0.2*
R-HSA-1592230	Mitochondrial biogenesis	0.2	0.2*
R-HSA-163200	Respiratory electron transport, ATP synthesis by chemiosmotic coupling, and heat production by uncoupling proteins.	0.265	0.265
R-HSA-6809371	Formation of the cornified envelope	0.269	0.269
R-HSA-1266738	Developmental Biology	0.278	0.278
R-HSA-1474228	Degradation of the extracellular matrix	0.288	0.288
R-HSA-9609507	Protein localization	0.332	0.332
R-HSA-1428517	The citric acid (TCA) cycle and respiratory electron transport	0.352	0.352
R-HSA-392499	Metabolism of proteins	0.358	0.358
R-HSA-196854	Metabolism of vitamins and cofactors	0.374	0.374
R-HSA-8878171	Transcriptional regulation by RUNX1	0.398	0.398
R-HSA-6805567	Keratinization	0.411	0.411
R-HSA-5673001	RAF/MAP kinase cascade	0.511	0.511
R-HSA-1852241	Organelle biogenesis and maintenance	0.518	0.518
R-HSA-71291	Metabolism of amino acids and derivatives	0.603	0.603
R-HSA-112316	Neuronal System	0.643	0.643
R-HSA-9012999	RHO GTPase cycle	0.667	0.667
R-HSA-212436	Generic Transcription Pathway	0.96	0.96
R-HSA-73857	RNA Polymerase II Transcription	0.971	0.971
R-HSA-74160	Gene expression (Transcription)	0.981	0.981
	**Excluded**		
R-HSA-9679506	SARS-CoV Infections	0	0.095
R-HSA-9679191	Potential therapeutics for SARS	0.007	0.001
R-HSA-164952	The role of Nef in HIV-1 replication and disease pathogenesis	0.068	0.097
R-HSA-9678108	SARS-CoV-1 Infection	0.135	0.135
R-HSA-9694516	SARS-CoV-2 Infection	0.17	0.17
R-HSA-162909	Host Interactions of HIV factors	0.295	0.295
R-HSA-5663205	Infectious disease	0.32	0.32
R-HSA-162906	HIV Infection	0.454	0.454

Supplementary Materials Table S4. Ontological categories for total NPI-Q.

*** only pathways with FDR<0.25 and p-value<were included for further analysis.**

Pathway identifier	Pathway name	p-value	False-discovery rate
**Immune response**			
R-HSA-6788467	IL-6-type cytokine receptor ligand interactions	0.012*	0.075*
R-HSA-6783589	Interleukin-6 family signaling	0.019*	0.077*
R-HSA-168256	Immune System	0.051	0.096*
R-HSA-977606	Regulation of Complement cascade	0.089	0.096*
R-HSA-166658	Complement cascade	0.096	0.096*
R-HSA-198933	Immunoregulatory interactions between a Lymphoid and a non-Lymphoid cell	0.187	0.187*
R-HSA-168249	Innate Immune System	0.195	0.195*
R-HSA-449147	Signaling by Interleukins	0.275	0.275
R-HSA-6798695	Neutrophil degranulation	0.287	0.287
R-HSA-1280215	Cytokine Signaling in Immune system	0.437	0.437
R-HSA-1280218	Adaptive Immune System	0.496	0.496
**Glycosylation and other posttranslational modifications**			
R-HSA-913709	O-linked glycosylation of mucins	0.044*	0.096*
R-HSA-5173105	O-linked glycosylation	0.076	0.096*
R-HSA-597592	Post-translational protein modification	0.65	0.65*
**Neurodegeneration**			
R-HSA-8863678	Neurodegenerative Diseases	0.015*	0.075*
R-HSA-8862803	Deregulated CDK5 triggers multiple neurodegenerative pathways in Alzheimer's disease models	0.015*	0.075*
**Response to stress**			
R-HSA-3299685	Detoxification of Reactive Oxygen Species	0*	0.009*
R-HSA-9711123	Cellular response to chemical stress	0.324	0.324
R-HSA-2262752	Cellular responses to stress	0.07	0.096*
R-HSA-8953897	Cellular responses to external stimuli	0.074	0.096*
**Other**			
R-HSA-9645723	Diseases of programmed cell death	0.048*	0.096*
R-HSA-5628897	TP53 Regulates Metabolic Genes	0.059	0.096*
R-HSA-3700989	Transcriptional Regulation by TP53	0.227	0.096*
R-HSA-212436	Generic Transcription Pathway	0.601	0.601
R-HSA-73857	RNA Polymerase II Transcription	0.637	0.637
R-HSA-74160	Gene expression (Transcription)	0.676	0.676
R-HSA-1643685	Disease	0.773	0.773
R-HSA-392499	Metabolism of proteins	0.783	0.783

Supplementary Materials Table S5. Ontological categories for depression.

***only pathways with FDR<0.25 and p-value< 0.05 were included for further analysis**

Pathway identifier	Pathway name	p-value	False-discovery rate
	**Lipid metabolism**		
R-HSA-8963901	Chylomicron remodeling	0.007*	0.055*
R-HSA-8963888	Chylomicron assembly	0.007*	0.055*
R-HSA-8963898	Plasma lipoprotein assembly	0.013*	0.055*
R-HSA-8963889	Assembly of active LPL and LIPC lipase complexes	0.013*	0.055*
R-HSA-8963899	Plasma lipoprotein remodeling	0.022*	0.055*
R-HSA-174824	Plasma lipoprotein assembly, Remodeling, and clearance	0.049*	0.055*
	**Immune reponse**		
R-HSA-168249	Innate Immune System	0.195	0.195*
R-HSA-6798695	Neutrophil degranulation	0.287	0.287
R-HSA-168256	Immune System	0.479	0.479
	**Glycosylation and other posttranslational modifications**		
R-HSA-9683686	Maturation of spike protein	0.004*	0.055*
R-HSA-879415	Advanced glycosylation endproduct receptor signaling	0.009*	0.055*
R-HSA-901042	Calnexin/calreticulin cycle	0.018*	0.055*
R-HSA-9694548	Maturation of spike protein	0.02*	0.055*
R-HSA-532668	N-glycan trimming in the ER and Calnexin/Calreticulin cycle	0.024*	0.055*
R-HSA-8957275	Post-translational protein phosphorylation	0.071	0.071*
R-HSA-446203	Asparagine N-linked glycosylation	0.192	0.192*
R-HSA-597592	Post-translational protein modification	0.649	0.649
	**Cell adhesion and signal transduction**		
R-HSA-418990	Adherens junctions interactions	0.022*	0.055*
R-HSA-421270	Cell-cell junction organization	0.044*	0.055*
R-HSA-446728	Cell junction organization	0.062	0.062*
R-HSA-8856825	Cargo recognition for clathrin-mediated endocytosis	0.07	0.07*
R-HSA-5576891	Cardiac conduction	0.084	0.084*
R-HSA-1500931	Cell-Cell communication	0.084	0.084*
R-HSA-983712	Ion channel transport	0.12	0.12*
	**Intra-cellular and cell transport**		

R-HSA-936837	Ion transport by P-type ATPases	0.037*	0.055*
R-HSA-382551	Transport of small molecules	0.086*	0.086*
R-HSA-8856828	Clathrin-mediated endocytosis	0.096*	0.096*
R-HSA-199991	Membrane Trafficking	0.363	0.363
R-HSA-5653656	Vesicle-mediated transport	0.419	0.419
	**Neurodegeneration**		
R-HSA-977225	Amyloid fiber formation	0.055	0.055*
	**Other**		
R-HSA-9683701	Translation of structural proteins	0.022*	0.055*
R-HSA-975634	Retinoid metabolism and transport	0.03*	0.055*
R-HSA-6806667	Metabolism of fat-soluble vitamins	0.033*	0.055*
R-HSA-9694635	Translation of structural proteins	0.033*	0.055*
R-HSA-5578775	Ion homeostasis	0.037*	0.055*
R-HSA-2187338	Visual phototransduction	0.067	0.067*
R-HSA-381426	Regulation of Insulin-like Growth Factor (IGF) transport and uptake by Insulin-like Growth Factor Binding Proteins (IGFBPs)	0.082	0.082*
R-HSA-196854	Metabolism of vitamins and cofactors	0.125	0.125*
R-HSA-397014	Muscle contraction	0.127	0.127*
R-HSA-9709957	Sensory Perception	0.35	0.35
R-HSA-1643685	Disease	0.403	0.403
R-HSA-392499	Metabolism of proteins	0.417	0.417
R-HSA-1430728	Metabolism	0.806	0.806
	**Excluded**		
R-HSA-9679506	SARS-CoV Infections	0.005	0.055
R-HSA-9678108	SARS-CoV-1 Infection	0.041	0.055
R-HSA-9694516	SARS-CoV-2 Infection	0.051	0.055
R-HSA-9679191	Potential therapeutics for SARS	0.055	0.055
R-HSA-5663205	Infectious disease	0.193	0.193

Pathway identifier	Pathway name	p-value	adjusted p-value
	**Lipid metabolism**		
R-HSA-8963901	Chylomicron remodeling	0.007	0.055
R-HSA-8963888	Chylomicron assembly	0.007	0.055
R-HSA-8963898	Plasma lipoprotein assembly	0.013	0.055
R-HSA-8963889	Assembly of active LPL and LIPC lipase complexes	0.013	0.055
R-HSA-8963899	Plasma lipoprotein remodeling	0.022	0.055
R-HSA-174824	Plasma lipoprotein assembly, Remodeling, and clearance	0.049	0.055
	**Immune reponse**		
R-HSA-168249	Innate Immune System	0.195	0.195
R-HSA-6798695	Neutrophil degranulation	0.287	0.287
R-HSA-168256	Immune System	0.479	0.479
	**Glycosylation and other posttranslational modifications**		
R-HSA-9683686	Maturation of spike protein	0.004	0.055
R-HSA-879415	Advanced glycosylation endproduct receptor signaling	0.009	0.055
R-HSA-901042	Calnexin/calreticulin cycle	0.018	0.055
R-HSA-9694548	Maturation of spike protein	0.02	0.055
R-HSA-532668	N-glycan trimming in the ER and Calnexin/Calreticulin cycle	0.024	0.055
R-HSA-8957275	Post-translational protein phosphorylation	0.071	0.071
R-HSA-446203	Asparagine N-linked glycosylation	0.192	0.192
R-HSA-597592	Post-translational protein modification	0.649	0.649
	**Cell adhesion and signal transduction**		
R-HSA-418990	Adherens junctions interactions	0.022	0.055
R-HSA-421270	Cell-cell junction organization	0.044	0.055
R-HSA-446728	Cell junction organization	0.062	0.062
R-HSA-8856825	Cargo recognition for clathrin-mediated endocytosis	0.07	0.07
R-HSA-5576891	Cardiac conduction	0.084	0.084
R-HSA-1500931	Cell-Cell communication	0.084	0.084
R-HSA-983712	Ion channel transport	0.12	0.12
	**Intra-cellular and cell transport**		

R-HSA-936837	Ion transport by P-type ATPases	0.037	0.055
R-HSA-382551	Transport of small molecules	0.086	0.086
R-HSA-8856828	Clathrin-mediated endocytosis	0.096	0.096
R-HSA-199991	Membrane Trafficking	0.363	0.363
R-HSA-5653656	Vesicle-mediated transport	0.419	0.419
	**Neurodegeneration**		
R-HSA-977225	Amyloid fiber formation	0.055	0.055
	**Other**		
R-HSA-9683701	Translation of structural proteins	0.022	0.055
R-HSA-975634	Retinoid metabolism and transport	0.03	0.055
R-HSA-6806667	Metabolism of fat-soluble vitamins	0.033	0.055
R-HSA-9694635	Translation of structural proteins	0.033	0.055
R-HSA-5578775	Ion homeostasis	0.037	0.055
R-HSA-2187338	Visual phototransduction	0.067	0.067
R-HSA-381426	Regulation of Insulin-like Growth Factor (IGF) transport and uptake by Insulin-like Growth Factor Binding Proteins (IGFBPs)	0.082	0.082
R-HSA-196854	Metabolism of vitamins and cofactors	0.125	0.125
R-HSA-397014	Muscle contraction	0.127	0.127
R-HSA-9709957	Sensory Perception	0.35	0.35
R-HSA-1643685	Disease	0.403	0.403
R-HSA-392499	Metabolism of proteins	0.417	0.417
R-HSA-1430728	Metabolism	0.806	0.806
	**Excluded**		
R-HSA-9679506	SARS-CoV Infections	0.005	0.055
R-HSA-9678108	SARS-CoV-1 Infection	0.041	0.055
R-HSA-9694516	SARS-CoV-2 Infection	0.051	0.055
R-HSA-9679191	Potential therapeutics for SARS	0.055	0.055
R-HSA-5663205	Infectious disease	0.193	0.193

Supplementary Materials Table S6. Ontological categories for apathy.

*** only pathways with FDR<0.25 and p-value<0.05 were included for further analysis.**

Participants without AD (n=41/15)				Participants with AD (n=7/21)	
A2AP	CO2	IBP6	PCOC1	ADIRF	PROC
ADA10	CO3	IBP7	PGRP2	B3GN2	PTHD2
AEBP1	CO5	IC1	PLMN	CD109	SODE
AFAM	CO8A	IPSP	PON1	CPVL	SUCO
AMBP	CRHBP	ITIH2	PPN	CRP	
ANGI	F13B	ITIH4	RARR2	K1C10	
APOA2	FA10	KAIN	RET4	K2C1	
APOA4	FBLN3	KNG1	RNAS4	KLKB1	
APOH	FETUA	LAMA2	SEP15	MSLN	
BMP1	FETUB	LG3BP	SEPP1	PRKDC	
C1RL	FIBA	LMAN1	SLPI		
CADH5	FMOD	LUM	SNED1		
CATH	GLDN	LYSC	TBD2A		
CBG	HABP2	MA1C1	THBG		
CBPN	HEG1	MA2A1	THRB		
CFAD	HELZ	MIA	VASN		
CFAH	HEMO	MYH15	VTDB		
CFAI	HEP2	MYH7	VTNC		
CL049	HRG	NID2	X1C12_P1		
CLUS	IBP3	OAF	ZA2G		

Supplementary Materials Table S7: Proteins differentially expressed in participants with or without NPS in participants without AD (left) or presenting cerebral AD (right). Numbers represent participants without NPS/participants with NPS (NPI-Q > 0).

	NPI-Q total score	NPI-Q depression	NPI-Q apathy	NPI-Q anxiety
mean	5.74	0.56	0.64	1.05
median	4	0	0	1
range	1-26	0-3	0-3	0-3

Supplementary Materials Table S8: **Mean, median and range for the NPI-Q total score and for depression, apathy and anxiety domains.**
